# Supplementary material for: Identifying biomarkers for diagnosis and disease activity monitoring in PSC-IBD and UC through proteomic profiling: A prospective, biomarker discovery single-center study protocol
Source: PLoS One. 2026 Jan 27;21(1):e0341885. doi: 10.1371/journal.pone.0341885 (PMC12843537; doi:10.1371/journal.pone.0341885)
Supplement: Attachment — Submitted filename: Reviewers_answers.docx [file pone.0341885.s003.docx]

We would like to thank the reviewers for their valuable comments. All suggestions and requests have been carefully considered, and the manuscript has been revised accordingly. Below, we provide our responses to each of the reviewers’ comments.

**Reviewer #1:** The study protocol, "Identifying biomarkers for diagnosis and disease activity monitoring in PSC-IBD and UC through proteomic profiling," is highly relevant. Still, its single-centre, discovery nature presents translational risks that must be mitigated.

Recommendations for the Protocol

*1) Validation and Generalisability:'Given that the study is single-centre, the protocol must explicitly acknowledge the need for future external validation' in an independent cohort. To strengthen the discovery phase, the statistical analysis plan should include a robust 'internal validation strategy', such as nested cross-validation, to prevent overfitting of the protein signature.*

Validation of the results from the discovery phase will be performed on external cohorts of cases, and this is explicitly stated in the Materials and Methods section, specifically under the subsection Study Setting and Timeline. (**pgs. 6-7, lines 131-137**)

In response to the reviewer’s request, the statistical section has also been expanded to provide a more detailed description of the validation process. (**pg. 10, lines 236-249**).

*2) Standardised Phenotyping: 'Biomarker results rely entirely on accurate clinical context. The protocol must mandate the use of the 'gold-standard measures' for defining disease states:
For UC activity and remission, use the 'Mayo Endoscopic Subscore' and a 'Histological Scoring System'(e.g., Nancy Index).
For PSC activity, use 'MRCP'(Magnetic Resonance Cholangiopancreatography) findings and serial 'serum Alkaline Phosphatase (ALP)' levels.*

We thank the reviewer for pointing out this aspect. The Mayo Score (clinical activity), the Mayo Endoscopic Subscore (endoscopic activity) and the Nancy Histological Index (histological activity) will be used for the assessment of the intestinal inflammation in both UC and PSC groups as described on **pg. 8, lines 190 – 191**. In the revised version of the manuscript, additional information has been included regarding the assessment of PSC cases using MRCP and ALP levels (**pg. 8, lines 191-193**).

*3) Clinical Endpoints: The predictive utility of any biomarker panel must be defined. The protocol should clearly state the 'long-term clinical outcomes' (e.g., treatment escalation, surgery, malignancy development) that the proteomic signatures will be tested to predict over the follow-up period.*

We thank the reviewer for this important comment. Since the primary aim of the study is to identify diagnostic biomarkers and markers that accurately reflect inflammatory activity, and both endoscopic and histological inflammatory activity are independent predictors of clinical outcomes, it can be assumed that the corresponding biomarker will also have predictive value for the potential development of future complications. Nevertheless, the authors plan to follow up this project with another study specifically focused on evaluating the predictive significance of the newly identified biomarkers. This follow-up study will include long-term patient monitoring and correlation of the biomarkers with the development of clearly defined long-term outcomes. This information has been added to the Study Setting and Timeline section of the manuscript (**pgs. 6-7, lines 131-137**).

**Reviewer #2**: In this study, Ondrej Fabian and colleagues aimed to identify novel proteomic biomarkers for PSC-IBD and UC by means of an untargeted proteomic analysis followed by multi-step validation in independent cohorts. The relatively large sample size (150 subjects in the discovery cohort) is commendable and addresses a common limitation in previous IBD proteomic studies. The study's focus on correlating proteomic profiles with disease phenotypes and histological activity is also promising, potentially leading to a more nuanced understanding of disease pathogenesis and improved diagnostic accuracy. The rationale for identifying more reliable biomarkers is clear: current diagnostic tools for IBD have limitations in accuracy and disease activity assessment, leading to delayed diagnoses and suboptimal treatment strategies.

*1. However, the study's clinical relevance could be further emphasized by explicitly addressing the shortcomings of currently available serological markers. While the study mentions assessing ASCA and pANCA, it needs to delve deeper into the limitations of these markers. For example, the lack of sensitivity and specificity that can overlap IBD or non-IBD conditions are key. More importantly, one critical point that should be recalled and discussed, is the frequent detection of ASCA in untreated celiac disease, as previously demonstrated (doi: 10.1111/j.1365-2036.2005.02417.x), suggesting that serum ASCA production might be a non-specific response to microbial antigens due to increased intestinal permeability. This phenomenon is particularly relevant as both celiac disease and IBD share similar clinical presentations and can sometimes be misdiagnosed, especially in early stages where both diseasorders may present ASCA positivity without clinical signs (Gut. 2006;55(2):296. ).
Therefore, the study should include a thorough discussion of these findings, emphasizing that ASCA positivity alone should not be considered a definitive marker for IBD, particularly in patients with atypical symptoms.*

We thank the reviewer for the relevant comment and for pointing out the related papers. A paragraph addressing the limitations of current serological markers has been added to the Discussion section, along with the relevant references. (**pgs. 13-14, lines 308-316**).

*2. The authors should clearly define the criteria used to differentiate PSC-IBD from UC and other forms of colitis. Given the phenotypic overlap, stringent diagnostic criteria are essential to ensure accurate biomarker identification.*

We agree entirely that precise diagnosis is an essential prerequisite of this type of study. In the Patients’ Eligibility Criteria and Recruitment section, it is stated that the diagnoses of PSC-IBD and UC were established according to the currently accepted international diagnostic criteria, which are supported by appropriate citations. However, at the reviewer’s request, this part of the text has been expanded, and the main differential diagnostic criteria for these diseases have been explicitly mentioned (**pg. 7 lines 146-158**).

3. *Longitudinal Assessment: While the study is prospective, it would be valuable to incorporate a longitudinal component to assess the biomarkers' ability to predict disease progression, response to therapy, and risk of complications.*

We acknowledge that longitudinal follow-up and evaluation of disease development are critical to the validity of potential biomarkers, and the first reviewer also emphasized the need for longitudinal evaluation. As mentioned in our previous response, since the primary aim of the study is to identify diagnostic biomarkers and markers that accurately reflect inflammatory activity, and both endoscopic and histological inflammatory activity are independent predictors of clinical outcomes, it can be assumed that the corresponding biomarker will also have predictive value for the potential development of future complications. Nevertheless, the authors plan to follow up this project with another study specifically focused on evaluating the predictive significance of the newly identified biomarkers. This follow-up study will include long-term patient monitoring and correlation of the biomarkers with the development of clearly defined long-term outcomes, i.e., need for therapy intensification, hospitalization, surgical intervention, development of Acute Severe Colitis, and the occurrence of dysplasia or invasive malignancy (**pgs. 6-7, lines 131-137**).

4. Cost-Effectiveness: The study should address the potential cost-effectiveness of the identified biomarkers in clinical practice. While novel biomarkers may improve diagnostic accuracy, their implementation depends on affordability and accessibility.

A paragraph on cost-effectiveness has been added to the end of the Discussion section (**pg. 15, lines 356-362**).

*5. Patient stratification. It might be good to mention the biomarkers that might help discriminate the disease behavior, and select different treatments for the patients.*

We thank the reviewer for the comment. The Discussion section has been expanded to include text highlighting the importance of biomarkers for improved patient stratification and for enabling more precise and targeted therapy (**pg. 13, lines 298-302**).
